# DLNR-SIQA: Deep Learning-Based No-Reference Stitched Image Quality Assessment

**DOI:** 10.3390/s20226457

**Published:** 2020-11-12

**Authors:** Hayat Ullah, Muhammad Irfan, Kyungjin Han, Jong Weon Lee

**Affiliations:** Mixed Reality and Interaction Lab, Department of Software, Sejong University, Seoul 143-747, Korea; hayat@sju.ac.kr (H.U.); irphan@sju.ac.kr (M.I.); kjinnhan@korea.ac.kr (K.H.)

**Keywords:** computer vision, deep learning, image quality assessment, image segmentation, immersive contents

## Abstract

Due to recent advancements in virtual reality (VR) and augmented reality (AR), the demand for high quality immersive contents is a primary concern for production companies and consumers. Similarly, the topical record-breaking performance of deep learning in various domains of artificial intelligence has extended the attention of researchers to contribute to different fields of computer vision. To ensure the quality of immersive media contents using these advanced deep learning technologies, several learning based Stitched Image Quality Assessment methods have been proposed with reasonable performances. However, these methods are unable to localize, segment, and extract the stitching errors in panoramic images. Further, these methods used computationally complex procedures for quality assessment of panoramic images. With these motivations, in this paper, we propose a novel three-fold Deep Learning based No-Reference Stitched Image Quality Assessment (DLNR-SIQA) approach to evaluate the quality of immersive contents. In the first fold, we fined-tuned the state-of-the-art Mask R-CNN (Regional Convolutional Neural Network) on manually annotated various stitching error-based cropped images from the two publicly available datasets. In the second fold, we segment and localize various stitching errors present in the immersive contents. Finally, based on the distorted regions present in the immersive contents, we measured the overall quality of the stitched images. Unlike existing methods that only measure the quality of the images using deep features, our proposed method can efficiently segment and localize stitching errors and estimate the image quality by investigating segmented regions. We also carried out extensive qualitative and quantitative comparison with full reference image quality assessment (FR-IQA) and no reference image quality assessment (NR-IQA) on two publicly available datasets, where the proposed system outperformed the existing state-of-the-art techniques.

## 1. Introduction

The recent rapid development of the field of virtual reality (VR) [1] has gained immense attention from researchers around the globe who have contributed to the VR community with new ideas and algorithms. These advancements in VR technologies have significantly developed simulation and interaction techniques for a variety of tasks including realistic battlefield simulations for military training [2], virtual assistance in production sectors [3], and enhancement of immersive and interactive user experience via advanced user interfaces. However, the performance of these advancements is heavily depending on the quality of the immersive contents that enable the users to view VR contents via freely moving inside the virtual world. These immersive contents are usually obtained by stitching multiple images captured through different cameras with varying viewpoints, overlapping gaps, and various lighting conditions, where the obtained stitched panoramic images suffer from various stitching errors [4,5].

**Figure 1:**
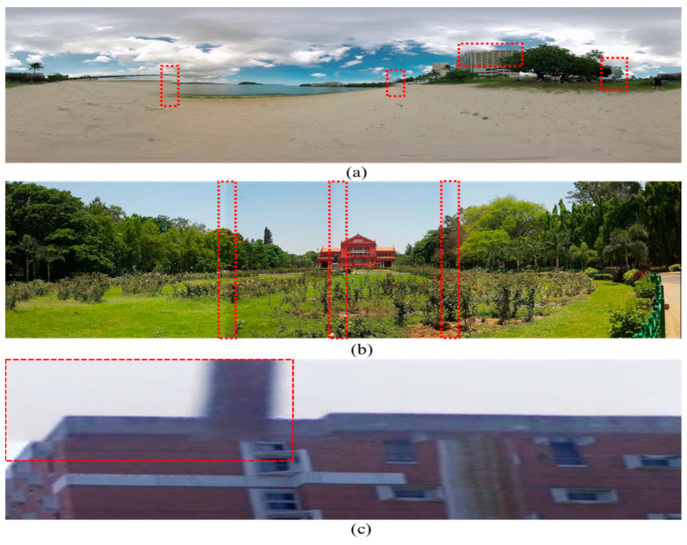
Illustration of various stitching errors: (**a**) Parallax error, (**b**) blending error, and (**c**) blur error.

One of the key advantages of the immersive contents experience is the wide field of view (FoV) perception, create with the help of panoramic images where a single wide-angle stitched image is produced from multiple smaller viewpoints images captured via various cameras [6,7]. The image stitching pipeline involves two main steps, such as geometric alignment and photometric correction. The Geometric alignment step computes the homography between adjacent images and performs image alignment based on the computed homography, where the photometric correction step is responsible for the color correction near the stitching region. Primarily stitching errors caused by the geometric alignment are due to the inaccurate measurement of the homographic transformation parameters that results in commonly observed stitching artifacts including parallax, blending, and blur errors, as shown in Figure 1, where the error specific regions are highlighted with red bounding boxes. In order to avoid such erroneous panoramic contents, the perceptual quality of the generated panoramic image must be assessed, and error-free images be selected for high quality immersive contents generation. However, the quality assessment panoramic contents based on these stitching errors is a very challenging task, especially when a single panoramic image contains numerous stitching errors. Each stitching error has their own impact on the quality of the panoramic/stitched image. For instance, parallax distortion disturbs pixel coordination, blending distortion introduces color variance near the stitching boundaries, and blur distortion reduces the visibility of panoramic contents. To better estimate the perceptual quality of the stitched image, these stitching errors be localized and analyzed based on their geometrical and photometrical properties. The geometric errors mostly occur due to inaccurate estimation of homography between two images, while the photometric errors are usually caused by the dissimilar lighting variations between two adjacent images.

Generally, the area of image quality assessment (IQA) has been actively researched in the last two decades, where a variety of methods are presented to assess the image quality. The early IQA approaches were focused on the quality of 2D images with different visual artifacts including Gaussian blur (BLUR) [8], JPEG compression (JPEG) [9], JPEG2000 compression (JP2k) [10], white noise (WN) [11], and fast fading (FF) [12]. These quality reduction artifacts have been assessed with both image fidelity metrics and learnable IQA methods. As for image fidelity metrics approaches, structural similarity index matrix (SSIM) [13], feature-similarity index matrix (FSIM) [14], peak signal-to-Noise ratio (PSNR) [15], and mean square error (MSE) [16] are used to measure the similarity between an original image and a distorted image. Besides these conventional image fidelity metrics, several learnable IQA models have been proposed [17,18,19,20] to predict image quality. For instance, Yan et al. [17] presented a multi-task CNN (Convolutional Neural Network) model to estimate the quality of an input image without any reference image. In their proposed model, first they computed natural scene statistics (NSS) and then predicted the image quality. Similarly, Liu et al. [19] proposed a deep-driven IQA method that focused on spatial dependency in the perceptual quality of an observed distorted image. Recently, Kim et al. [20] presented a receptive field generation-oriented IQA approach that performs image quality estimation in two steps. In the first step, receptive fields are generated from the given distorted image. Next, the generated receptive fields and visual sensitivity maps are utilized to weight the visual quality of the observed image. Despite providing promising performance in terms of quality estimation, these methods are still limited to 2D IQA tasks and unable to capture the stitching artifacts in panoramic images., Since stitching artifacts are more complex and eye-catching as compared to conventional artifacts in 2D images, which greatly reduces the overall quality of a stitched image.

To specifically assess the visual quality of panoramic images, numerous stitched image quality assessment (SIQA) methods have been presented in the past decade. Among the diversity of the stitching literature, a number of various researchers focus on the quality assessment of the stitched images by either using conventional handcrafted features based [21,22] methods or making subjective comparisons [23,24,25]. Broadly, the area of stitch image quality assessment (SIQA) is different from classical IQA in two perspectives. Firstly, the panoramic stitched images mostly suffer from geometric errors such as shape breakage and objects displacement, whereas classical IQA techniques are unable to assess the image quality. Secondly, unlike classical image distortions, stitching errors are local distortions including color seams near the stitching boundary, blur, and parallax error. The subjective SIQA methods [23,24,25,26,27] involve user studies where users are provided with a set of images and are asked to assign a quality score to each image. The participants analyze the given panoramic image in an HMD (Head Mounted Device) device in detail and assign a quality score to each image based on the visual quality of panoramic contents. Although subjective SIQA methods are very accurate in terms of quality prediction, these methods are expensive, time consuming, and difficult to use in practical applications. In addition, these methods have poor consistency because user opinion about image quality varies from person to person. On the other hand, objective SIQA methods [22,28,29,30] automatically estimate and predict the perceptual quality of given images using computer vision algorithms. These objective SIQA approaches take stitched images as an input and extract pixel-level information near the stitched regions. The extracted features can be used to predict the quality of stitched images. The objective SIQA methods are further classified into two classes: FR-SIQA (Full-Reference SIQA) and NR-SIQA (No-Reference SIQA) methods. The FR-SIQA methods usually take two input images: (1) a distorted stitched image and (2) a reference image, where the distortion-free reference image provides additional detail for evaluating the perceptual quality of the distorted stitched image. In contrast, NR-SIQA methods predict the quality of stitched images without any reference image. Instead of computing the similarity between a distorted stitched image and reference distortion-free image, NR-SIQA methods exploit different image properties, namely chrominance, structural consistency, histogram statistics of stitched image, and visibility of panoramic contents. The coming subsections presents the detailed literature review of state-of-the-art methods of the FR-SIQA and NR-SIQA domains, respectively.

### 1.1. Full-Reference Stitched Image Quality Assessment

The early objective SIQA work was based on FR-SIQA methods, where they estimated the perceptual quality of the given stitched images using image fidelity metrics in the presence of distortion-free reference images. For example, Yang et al. [31] proposed a content-aware SIQA method that captured the ghosting and structure inconsistency errors in panoramic images. Their proposed technique estimated the perceptual quality of the given stitched image in two steps. First, they estimated the local variance of optical flow field for reference images and distorted stitched images. In the second step, they computed the intensity and chrominance gradient of both pairs of images in highly structured patches. Finally, the outputs of both error estimation modules (ghosting and structure inconsistency) are combined, and the weighted perceptual quality score is predicted. To form a unified SIQA metric, they combined these measures using an optimally weighted linear combination. Zhou et al. [32] presented a two-couple feature point matching-based approach for the quality estimation of urban scenery stitched images. They used image fidelity metrics including SSIM and high frequency information SSIM (HFI-SSIM) to estimate the difference between distorted stitched images and reference images. Similarly, Li et al. [21] proposed an omnidirectional image quality assessment framework that estimates the perceptual quality of omnidirectional contents. While estimating the quality of the stitched image, they used 0° and 180° as a target and 90° and 270° as cross-reference regions. The target stitched regions are then assessed by exploiting the relationship between target and reference stitched regions using perceptual hash, sparse reconstruction, and histogram statistics. Yan et al. [22] proposed a perceptual quality estimation metric for stereoscopic stitched images that captured common stitching errors including color distortion, structure inconsistency, ghost distortion, and disparity distortion. For quality estimation in the presence of these distortions, they used information loss, points distance, color difference coefficient, matched line inclination degree, and disparity variance. Although these FR-SIQA methods are fast and accurate, it is usually difficult and sometimes impossible to have panoramic reference images in advance. Due to the requirement of huge amounts of reference image data, these methods are limited to subject quality assessment of panoramic images and unable to assess the quality of a panoramic image without a reference image.

### 1.2. No-Reference Stitched Image Quality Assessment

Recently, several NR-SIQA methods [33,34,35,36,37] have been proposed to automate the SIQA process. These methods estimate the perceptual quality of a given stitched image without using any stimulus information. For example, in [33], the authors introduced a convolutional sparse coding (CSC) technique to learn the pattern of stitching relevant distortion in a target image. They used different sets of convolution filters to localize the distortion region and, later, quantified the compound effect of these localized distortions using trained kernels. Madhusudana et al. [34] presented a steerable pyramid decomposition framework that estimated the perceptual quality of stitched images. Their proposed method used a gaussian mixture model and bivariate statistics to capture the ghosting, blur, and structure inconsistency in panoramic images. However, the performance of their system is limited for the color image distortion. To evaluate the visual quality of omnidirectional images, Li et al. [35] proposed an attention-driven omnidirectional IQA framework. Their work is focused on the perceptual quality of stitching regions and attention regions, where they used both local and global metrices to inspect those regions for stitching artifacts, color distortion, and resolution of stitched regions. Sun et al. [36] presented a learning-based framework for a no-reference 360 IQA using a multi-channel CNN. Their proposed method consists of two individual modules including a multi-channel CNN architecture followed by a regressor, where a CNN architecture extracts discriminative features from the intermediate layer and the image quality regressor processes the extracted features and predicts the quality score. Xu et al. [37] presented a learning based approach called Viewport-oriented Graph Convolutional Neural Network (VGCN) to estimate the perceptual quality of omnidirectional images. Inspired by the human vision system (HVS), first a spatial viewport graph was created to select a viewport with higher probabilities. Next, they used a graph convolutional network to perform reasoning on their proposed viewport selection graph. Finally, they obtained the global quality of omnidirectional images using the selection viewpoint and viewing experience of the user. These NR-SIQA methods are more realistic than FR-SIQA approaches and can predict the perceptual quality of panoramic contents. However, these methods are not consistent for a certain type of stitching error and some are focused on geometric distortions, while other studies examined photometric errors. In addition, [33,34,37] used computationally expensive procedures to capture stitching-specific distortions that are unable to localize specific distortions. The localization of stitching-relevant distortion can greatly improve the SIQA performance and compute the weighted magnitude of each distortion. To address these issues in the existing SIQA methods, we introduce a learning-based NR-SIQA framework that first segments stitching distortion (i.e., parallax, blending, and blur) and then extracts specific distorted regions from the panoramic image. The proposed framework estimates the perceptual quality of stitched images using extracted distorted regions. To this end, the main contribution of this paper can be summarized as follows:

- Visually assessing the quality of 360° images is a very challenging problem where the existing SIQA approaches use deep features and a regressor model to find only the final score of the immersive images. To address this problem, we propose a novel three-fold DLNR-SIQA framework to localize stitching errors and recognize the type of errors present in the 360° images.
- To localize and find the type of stitching error present in the panoramic 360° images, we fine-tuned a Mask R-CNN [38] network on a publicly available Google Street View dataset. In the dataset, various types of stitching errors are manually annotated where the Mask R-CNN is retrained on the annotated data to localize and classify the stitching distortions.
- We develop a post-surgery technique that efficiently extracts specific distorted regions from the panoramic contents. The extracted information is then further analyzed to assess the essential characteristics of each distorted region, for example, the number of distorted pixels that help the image quality estimation module to measure the optimal perceptual quality. Further, we conduct extensive experiments on two benchmark SIQA datasets, where the obtained quantitative and qualitative results demonstrated the effectiveness of the proposed DLNR-SIQA framework against the existing SIQA methods.

The rest of this article is arranged as follows. Section 2 explains the major components of the proposed framework. A detailed experimental evaluation and comparative analysis of our framework is given in Section 3. Finally, this article is concluded in Section 4 with possible future directions.

## 2. Proposed Framework

**Figure 2:**
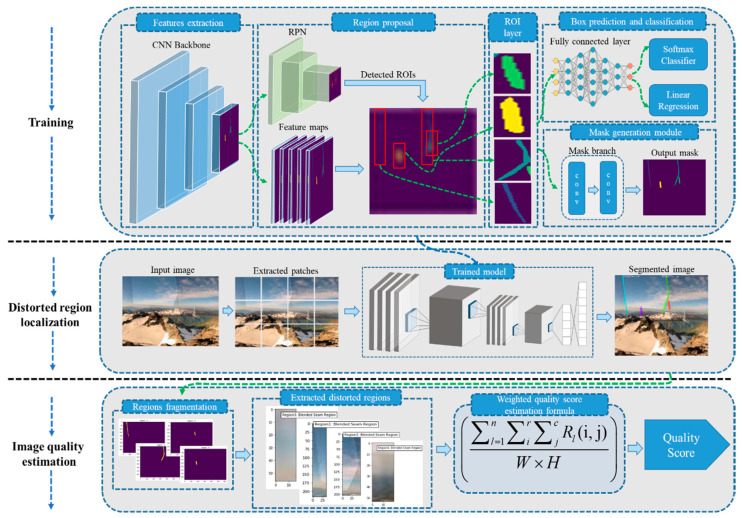
A detailed overview of our proposed DLNR-SIQA framework for stitching distortion localization and image quality estimation, which involves three main steps: training, distortion region localization, and image quality estimation. Step 1: The training processing procedure of the Mask R-CNN is demonstrated. Step 2 involves the segmentation of stitching distortions, where an input panoramic image is first converted into a set of patches and individual patches are forwarded to the fine-tuned Mask R-CNN. The output of the distortion region localization phase is a segmented distorted panoramic image, which is then forwarded to Step 3, where each segmented region is investigated individually and the perceptual quality is estimated by estimating the total distorted area over the total area of the panoramic image.

To the best of our knowledge, there is no single SIQA method that has examined the characteristics of individual stitching errors. With these motivations, we propose a learning-based NR-SIQA framework in this paper that first analyzes the individual stitching error and then obtains a weighted quality score by fusing the ratio of all errors. For better understanding, the proposed framework is divided into three main phases: (1) finetuning Mask R-CNN, (2) localization of distorted region, and (3) image quality estimation. The proposed framework along with technical components are illustrated in Figure 2.

### 2.1. Fine-Tuning Mask R-CNN for Stitching Distortion Segmentation

Lately, numerous CNN-assisted approaches have been proposed for a variety of applications, including activity recognition [39,40], video summarization [41,42], autonomous vehicle [43,44], and disaster management applications [45,46]. Considering the generalization and strength of CNNs in various research areas, in this paper, we proposed a Mask R-CNN-based solution to segment the distorted regions in panoramic stitched images. A detailed overview of Mask R-CNN architecture is given in Section 2.1.1, while the model training and loss function is explained in Section 2.1.2.

#### 2.1.1. Overview of Mask R-CNN Architecture

Mask R-CNN was originally introduced as a generic framework for object localization and object instance segmentation in natural images [38]. The standard Mask R-CNN has been derived from the Faster R-CNN [47] architecture by adding a new branch called a mask branch in parallel with bounding box prediction and a classification branch at the tail of the network. The extended Mask R-CNN has the ability to detect, segment and generate high quality masks for each segmented region. Due to easy adaptation, Mask R-CNN is used for variety of computer vision tasks and has obtained reasonable results. The Mask R-CNN architecture consists of three major components: a backbone feature pyramid network (FPN), region proposal network, and ROI selection followed by bounding box recognition and mask prediction modules. The selection of an efficient backbone network for the feature extraction phase is a challenging step, where the complexity of the network is greatly related to the behavior of training data. We are targeting stitching distortions in panoramic stitched images and the structures of these distortions have irregular boundaries that require a robust feature representation network. Having a deep hierarchical nature with multi-scale characteristics, a residual neural network (ResNet) [48] is the best candidate for the backbone feature extractor. Our proposed method adopts both ResNet-50 and ResNet-101 in individual training stages and evaluates the performance of Mask R-CNN with both architectures for training and testing, respectively. The backbone CNN architecture takes a distorted stitched patch as an input and extracts patch-level discriminative features at different scales. The extracted feature maps have shaded representations of distorted regions which are then forwarded to the Region Proposal Network (RPN) module. The RPN module scans the input feature maps with a sliding window to capture the ROI with stitching distortion. In the initial stages, RPN roughly generates a cluster of anchors (regions covered by sliding windows) with different aspect ratios and sizes. The roughly estimated anchors are then inspected by the RPN regressor where the best candidate anchors with the highest foreground scores are selected. After the region proposal process, selected anchors are then propagated to the ROI align layer which adjusts the alignment and spatial dimensionality of all selected anchors. Finally, the processed anchors are forwarded to two different submodules: (1) a bounding box recognition (prediction and classification) module and (2) mask generation module. The bounding box recognition module processes the input features using fully connected layers and forwards the processed features to the regression and classification head. The regression head predicts the final coordinates of the bounding box for each ROI where a classification head classifies the target category inside the ROI area. On the other hand, instead of fully connected layers, the mask generation module contains a CNN network called a mask branch. The mask branch generates binary mask from the ROI aligned feature maps. The overall flow of a typical Mask R-CNN is shown in Figure 2 (Training module).

#### 2.1.2. Model Training and Loss Function

To train the network, we used the existing open-source implementation of Mask R-CNN implemented by Matterport, Inc. [49]. The original network was trained on a benchmark common objects in context (COCO) dataset [50] widely used for object detection, object instance segmentation, and super pixel stuff segmentation. To fine tune the Mask R-CNN on our dataset, we select distorted stitched images from the Google Street View dataset [51] and the LS2N IPI (Image Perception Interaction) Stitched Patched dataset [33]. We collected a total of 1370 distorted patches from both datasets and divided them into training and validation sets with a split ratio of 70% and 30%, respectively. To meet the input dimensionality requirement of the network, all the images are cropped to m×*n*×c image size, where m = 256, *n* = 256 and c = 3. Before training, we manually annotated both training and validation data, where we selected the exact coordinates of the stitching distortions using an online annotation tool called VGG (Visual Geometry Group) Image Annotator (VIA). Our proposed framework was trained with two different backbone CNN architectures, ResNet50 and Resnet101. During training, Mask R-CNN used a joint loss function for distortion classification, bounding box regression, and mask prediction, respectively. Mathematically, the joint loss function can be expressed as follows:

(1) $$L=\ell_{class}+\ell_{bbox}+\ell_{mask}$$

Here, $\ell_{class}$ is the classification loss, $\ell_{bbox}$ is the bounding box regression loss, and $\ell_{mask}$ indicates the mask prediction loss. The classification loss can be computed by:

(2) $$\ell_{class}=\frac{1}{\eta_{class}}\sum_{i}-\log\left[{p^{\prime }}_{i}p_{i}+(1-{p^{\prime }}_{i})(1-p_{i})\right]$$

Here, $\eta_{class}$ indicates the number of the class, $p_{i}$ is the predicted probability of the *i*th ROI, whether it is predicted as positive (foreground) or negative (background). Where ${p^{\prime }}_{i}$ is the ground truth probability of *i*th ROI, the ground truth value for positive ROI (foreground) is 1, while for negative ROI (background), the ground truth value is 0. The computation of bounding box regression loss can be expressed as follows:

(3) $$\ell_{bbox}=\frac{1}{\eta_{nop}}\sum_{i}\left[{p^{\prime }}_{i}R(t_{i},{t^{\prime }}_{i}\right)]$$

where, $\eta_{nop}$ indicates the total number of pixels in the observed feature map, and *R* is the smooth L1 loss function commonly used for bounding box regression with less sensitivity for outlier regions. Mathematically, the *R* function can be expressed as follows:

(4) $$R(t_{i}{}^{\prime },t_{i})=smooth_{L1}(t_{i}{}^{\prime }-t_{i}),\;smooth_{L1}(x)=\left\{ \begin{array}{l} 0.5x^{2}\;\mathrm{if}\left|x\right|<1\\ \left|x\right|-0.5\;otherwise \end{array}\right.$$

Here, *t_i_* holds the difference between the four coordinates (including horizontal coordinate, vertical coordinate, width, and height) of the predicted anchor/bounding box and ground truth bounding box, where *t_i_*^’^ represents the difference between ground truth bounding box and the positive bounding boxes. Furthermore, the mask prediction loss can be computed by:

(5) $$\ell_{mask}=-\frac{1}{m^{2}}\sum_{1\leq (i,j)\leq m}\left[y_{\left(i,j\right)}^{k}=(1-y_{\left(i,j\right)})\log(1-y_{\left(i,j\right)}^{2})\right]$$

Here, $m^{2}$ is the m×m distorted region, $y_{\left(i,j\right)}$ is the ground truth label of the pixel at the (*i*,*j*) location in the distorted region, and $y_{\left(i,j\right)}^{k}$ is the predicted label of the pixel at the (*i*,*j*) location for the *k*th class. For instance, $y_{\left(i,j\right)}^{0}=1$ indicates the misclassification of the background pixel as foreground class, while $y_{\left(i,j\right)}^{1}=1$ represents the correct classification of the foreground pixel. Similarly, $y_{\left(i,j\right)}^{0}=0$ indicates the correct classification of the background pixel, while $y_{\left(i,j\right)}^{1}=0$ represents the misclassification of the foreground pixel.

### 2.2. Distorted Region Segmentation and Mask Generation

In this phase, we deployed a fine-tuned trained Mask R-CNN for segmenting distortions in stitched images. The panoramic stitched images have a wider FOV compared to normal 2D images, which cannot be input to the proposed network in the original resolution. Therefore, before forwarding to the network, we fragmented the high-resolution panoramic image into 128 patches with a dimensionality of m × *n* × c, where, m = 256, *n* = 256 and c = 3. The finetuned Mask R-CNN takes a panoramic stitched image as a batch of patches, where each patch is processed as an individual image. During distortion segmentation, the trained network traverses each patch for stitching distortion and captures the location of distorted regions. The captured locations of distorted regions are then enhanced by processing them at multiple convolutional layers of the generate binary masks for each captured distorted region. Finally, all processed patches are merged together and form a final segmented image, where each distorted region is specified by a separate binary mask.

### 2.3. Image Quality Estimation

The image quality estimation module is responsible for the perceptual quality estimation of the segmented stitched image. The proposed mechanism of image quality estimation involves three steps: region fragmentation, extraction of the distorted region from the original image using the fragmented region, and average distorted area in the stitched image. Each step of the proposed image quality estimation mechanism is explained in Algorithm 1. The first step fragments the binary mask map of a received segmented image into multiple mask maps and fragmentation is performed so that each fragmented mask map contains the mask of an individual distorted region. The fragmentation process facilitates the proposed system to individually investigate each distorted region in a separate mask map, thereby providing ease for the next module to process the fragmented mask maps in a more efficient way. The second step extracts the distorted regions from the original stitched image using fragmented mask maps. During the region extraction phase, we first estimate the contour of each distorted region using the corresponding mask. The computed contours are then used to extract the distorted regions from the original image. In the last step, the extracted regions are forwarded to average the distorted area estimation module, which calculates the area of individual distorted regions.
**Algorithm 1: Quality Estimation of Stitched Image****Input**: S*_i_* = Segmented Image**Output**: Quality Score Q*_s_***Prepossessing:****Steps:**1: Read the segmented image and perform regions fragmentation using binary masks.  Fragment*_i_* = image_fregmentation (S*_i_*)2: Extract the distorted region using fragmented regions.  Region*_i_* = region_extraction (Fragment*_i_*)3: Compute the pixel wise ratio of distortion-free image area.  Q_*s*_ = $\left(\frac{\sum_{l=1}^{n}\sum_{i}^{r}\sum_{j}^{c}R_{l}(i,j)}{W\times H}\right)$


The area of each extracted distorted region is computed one after another and added together. Finally, the target image quality score is obtained by dividing the total distorted area by the total area of the stitched image. Mathematically, the average distorted area estimation module can be expressed as:

(6) $$QS=\left(\frac{\sum_{l=1}^{n}\sum_{i}^{r}\sum_{j}^{c}R_{l}(i,j)}{W\times H}\right)\times 100$$

Here, *R_l_* is the lth region, *i* and *j* represent the *i*th row and *j*th column of a specific region; similarly, *W* and *H* are the corresponding width and height of the patch.

## 3. Experimental Results and Discussion

**Figure 3:**
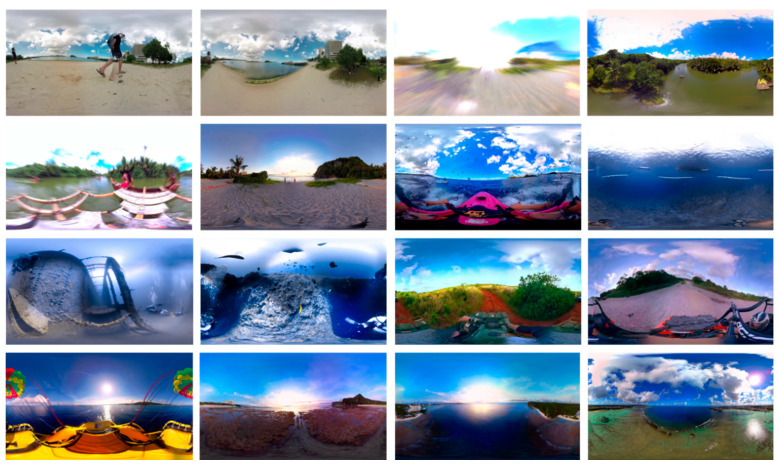
Representative distorted panoramic images from test set B.

**Figure 4:**
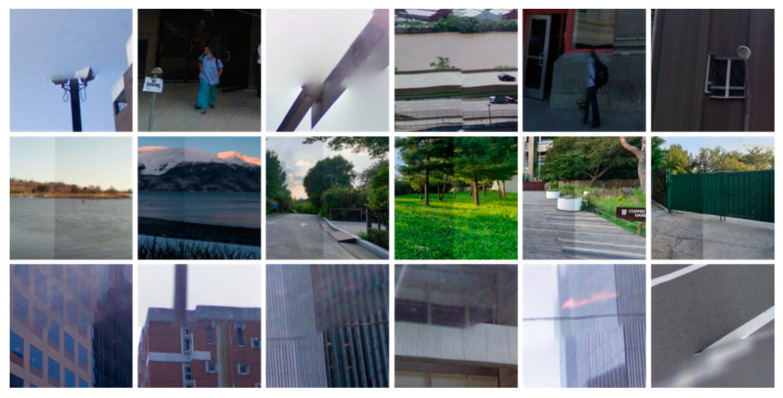
Representative distorted stitched patches from Google Street View Dataset [49] in test set A.

In this section, we present a detailed experimental evaluation of the proposed framework, both quantitatively and qualitatively. For quantitative perspective, we used different segmentation performance evaluation metrics including Precision (P), Recall(R), Dice (DSC), Jaccard Index (JI), Mean Pixel Accuracy (mPA), Mean Average Precision (mAP) and Mean Absolute Error (mAE). For qualitative evaluation, the obtained segmentation masks, distortion-specific regions and final segmented images are visually inspected. For experimental evaluation, we used two test sets: the patches test set (test set A) and the panoramic images test set (test set B) from the Google Street View Dataset [51] and the SUN360 Dataset [52], respectively. The test set A consists of 300 distorted stitched patches of size 256 × 256 × 3, test set B comprises 160 panoramic images of size 4096 × 2048 × 3. During the segmentation process, each panoramic image is first divided into 128 patches, where we conduct a series of experiments on different patch sizes and choose the optimal size for patch extraction. The statistical details of both test sets are listed in Table 1, whereas the representative samples of both patches test sets and panoramic images test sets are depicted in Figure 3 and Figure 4, respectively. Furthermore, we evaluated the performance of the fine-tuned Mask R-CNN with two different backbone architectures, i.e., ResNet-50 and ResNet101.

### 3.1. Experimental Details

The proposed DLNR-SIQA framework was implemented using Python version 3, Tensorflow and Keras on a machine. The training and experimental evaluation of our proposed framework was performed on a PC with the following hardware specifications: Nvidia GTX 1060 GPU (6 GB), 3.3 GHz processor, and 8 GB onboard memory. The proposed training strategy adopted two main modifications in the original implementation of Mask R-CNN [49]. (1) Rather than training the complete network from the very first layer, we squeezed the rest of the layers and trained only the network head by using the already learned weights of the COCO dataset. (2) We modified the hyper-parameters for fine tuning the Mask R-CNN on our custom stitched images dataset. The fine-tuned Mask R-CNN was trained for 50 epochs using an Adam optimizer with 100 training steps per epoch, a batch size of eight, a learning rate of 0.0001, and a momentum of 0.9.

### 3.2. Quantitative Evaluation

In this section, we present the quantitative evaluation of our proposed framework on two different types of images, i.e., stitched patches from test set A and panoramic images from test set B. The proposed quantitative evaluation protocol contains a set of metrices that are commonly used for estimating object instance segmentation performance, i.e., P, R, DSC, JI, mPA, mAP and mAE. The first two evaluation metrics P and R are used to evaluate the per-pixel binary mask prediction performance of our proposed DLNR-SIQA. Mathematically, P and R can be expressed by:

(7) $$P=\frac{TP}{TP+FP}$$

(8) $$R=\frac{TP}{TP+FN}$$

**Figure 5:**
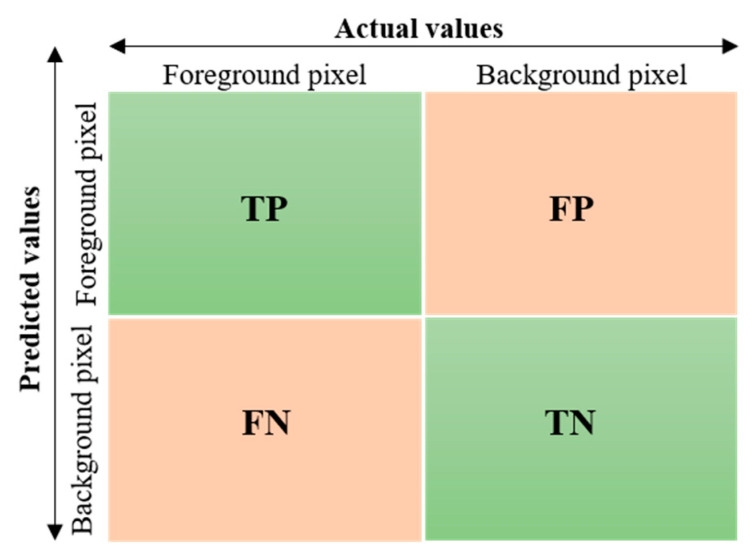
Confusion matrix for binary classification of segmented pixels.

Here, *TP* represents a group of pixels that are foreground pixels and also predicted as foreground pixels, FP represents a group of pixels that are background pixels but predicted as foreground, and the term *FN* represents a group of pixels that are foreground pixels but predicted as background, as shown in Figure 5. To estimate the similarity between the predicted segmentation mask and the ground truth mask, DSC and *JI* are used as evaluation metrics. Mathematically, DSC and JI can be expressed by:

(9) $$DSC=\frac{2\left|G_{SM}\cap P_{SM}\right|}{\left|G_{SM}\right|+\left|P_{SM}\right|}$$

(10) $$JI(G_{SM},P_{SM})=\frac{\left|G_{SM}\cap P_{SM}\right|}{\left|G_{SM}\cup P_{SM}\right|}$$

Here, GSM and PSM are the ground truth and predicted segmentation mask, respectively. The values of both DSC and JI vary from 0 to 1, where high values indicate better segmentation performance while low values indicate worse segmentation performance. To estimate the percentage of correctly classified pixels per segmentation mask, we used a well-known segmentation evaluation metric called mPA. Mathematically, mPA can be expressed by:

(11) $$mPA=\frac{1}{c}\sum_{i=0}^{c}\frac{P_{ii}}{\sum_{j=0}^{c}P_{ij}}$$

Here, *c* is the number of classes including the background, and *P_ii_* is the total number of pixels that are correctly classified, where *P_ij_* indicates misclassified pixels. Furthermore, we examined the performance of our method using *mAP* and *mAE* metrics, which are commonly used for object detection and segmentation performance evaluation. Average precision (*AP*) represents the amount of area under the precision–recall curve, where *mAP* can be obtained by computing the mean of *AP* over the total number of classes/categories.

(12) $$mAP=\frac{1}{n}\sum_{i=1}^{n}AP_{i}$$

Here, *AP* is the average precision, and *n* is the total number of classes. On the other hand, *mAE* calculates the absolute difference between pixels of the predicted segmentation mask and corresponding ground truth segmentation mask. Mathematically, *mAE* can be expressed by:

(13) $$mAE=\frac{\sum_{i=1}^{n}\left|y_{i}-x_{i}\right|}{n}$$

**Figure 6:**
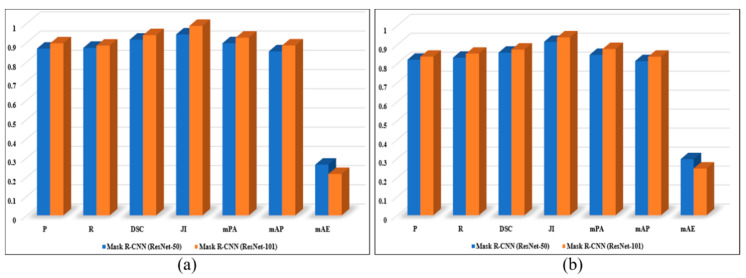
Quantitative performance of our proposed framework for: (**a**) stitched patches test set; (**b**) 360° images test set.

Here, *y_i_* is the *i*th pixel of the predicted segmentation mask, *x_i_* is the ith pixel of the ground truth segmentation mask, and *n* indicates the total prediction made by the network. The obtained results from quantitative evaluation for the stitched patches test set and 360° image test set are depicted in Figure 6.

### 3.3. Qualitative Evaluation

**Figure 7:**
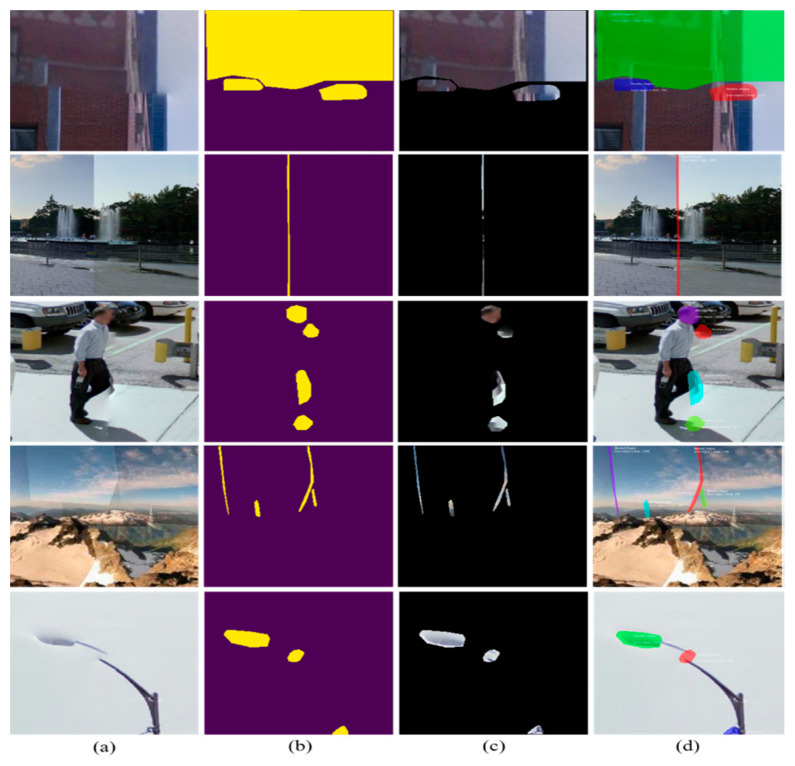
The segmentation results obtained for the distorted stitched patches test set: (**a**) input distorted patch; (**b**) generated mask map; (**c**) distortion-specific patch; and (**d**) final segmented images.

**Figure 8:**
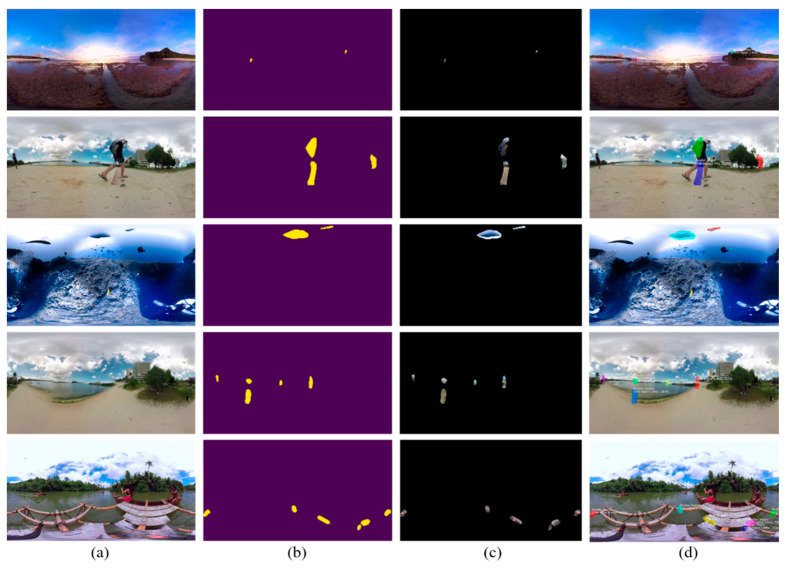
The segmentation results obtained for the distorted panoramic images test set: (**a**) input distorted panoramic image; (**b**) generated mask map; (**c**) distortion-specific panoramic image; (**d**) final segmented panoramic image.

Besides quantitative evaluation, we further evaluated the qualitative performance of our proposed framework by visually inspecting the segmentation masks obtained and the final segmented distorted images. To assess the generalization of our stitching distortion segmentation framework, we validated the proposed framework with two different types of stitched images, i.e., stitched patches and panoramic 360° images. For stitched patches, we selected distorted stitched patches from a Google Street View dataset [51]. The proposed framework processes the input patches in several stages (including feature extraction, ROI selection, ROI alignment, box prediction-classification, and mask generation) and returns two outputs for each input, i.e., binary mask and final distortion segmented image. The visual results obtained for stitched patches and full panoramic images are shown in Figure 7 and Figure 8, where the first column represents the input images, the second column represents the generated mask maps, the third column represents the distortion-specific images, and the last column represents the final segmented images.

### 3.4. Distorted Region Extraction and Quality Estimation

**Figure 9:**
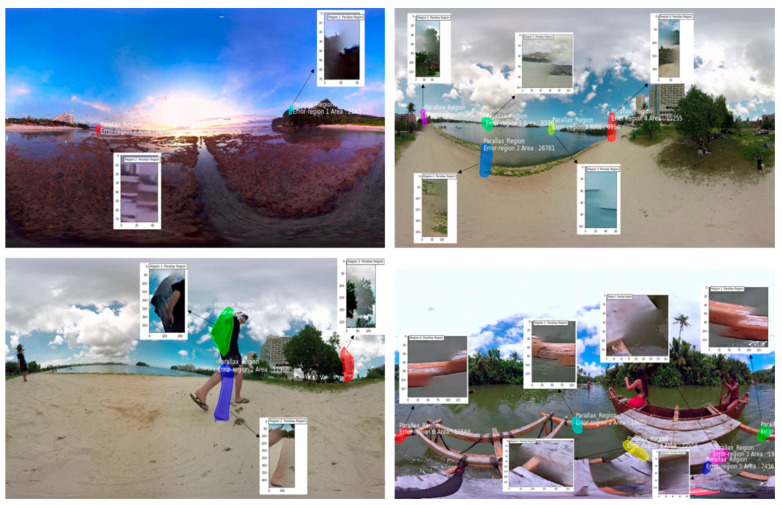
Extraction and visual inspection of segmented distorted regions in panoramic images.

After the segmentation of stitching distortions in panoramic images, we extracted the segmented distortion-specific regions from panoramic images using their corresponding masks. For each distorted region, we used the binary mask pixel value and selected the segmented area pixel from the original RGB image as shown in Figure 9. The extracted distorted regions were then used for the quality estimation of panoramic images. The perceptual quality of a given panoramic image was estimated using our own quality estimation scheme where we assessed the quality of panoramic images by computing the number of distorted pixels, the number of distortion-free pixels, the ratio of distortion, and the ratio of the distortion-free panoramic image. For this purpose, we first calculated the number of pixels for each distorted region. Next, the calculated pixels for all distorted regions were combined and divided by the total number of pixels of the original image, as given in Equation (5). The estimated perceptual quality of stitched patches and 360° panoramic images are given in Table 2, where the second and third columns list the values of distorted and distortion-free pixels, while the fourth and fifth columns list the percentage of distorted and distortion-free images. Using simple and pixel-level assessments of panoramic images, the proposed method provides an accurate estimation of perceptual quality, thereby exploiting the disturbance of pixels only in distortion-specific regions rather than traversing the entire panoramic image.

### 3.5. Comparison of Our Proposed Method with State-Of-The-Art SIQA Methods

In order to validate the effectiveness and generalization of the proposed DLNR-SIQA framework, we conducted a comparative analysis with existing deep learning-based FR-SIQA and NR-SIQA approaches [31,33,34]. The comparison was performed on two publicly available stitched images datasets: the SIQA [31,33] and the ISIQA (Indian Institute of Science Stitched Image QA) [34] dataset. The proposed framework is compared with the existing SIQA methods using three standard metrics including SRCC (Spearman’s Rank Correlation Coefficient), PLCC (Pearson’s Linear Correlation Coefficient), and RMSE (Root Mean Square Error). The metric SRCC estimates prediction similarity, while PLCC and RMSE estimates the prediction accuracy. The high value of SRCC and PLCC indicates the better performance where the lower RMSE reflects the better performance. Since the proposed framework is trained on images with three types of stitching distortion, parallax, blending, and blur distortion, we selected only those images that contained the aforementioned stitching distortions. Moreover, for the performance assessment of our proposed method along with other comparative methods, and furthermore, to obtain better correlation between MOS values and the objective scores predicted by the models, we followed the strategy of [37] by utilizing the five parameter logistic function:

(14) $$y=\beta_{1}\left(\frac{1}{2}-\frac{1}{1+\exp(\beta_{2}(x-\beta_{3}))}\right)+\beta_{4}x+\beta_{5}$$

where variable *x* indicates the prediction made by objective models and variable *y* represents the corresponding MOS score. Further, variables *β*_1_ to *β*_5_ are the controllable parameters to optimize the logistic function. To emphasize the effect of the logistic function, we evaluated the performance of our method with and without the use of the logistic function. The obtained results from the conducted experimental study on both the SIQA and ISIQA datasets are listed in Table 3. From the results, it can be perceived that the proposed method without the logistic optimization function dominated [31] in terms of the SRCC, PLCC, and RMSE on SIQA datasets; however, it performed better [33], obtaining the lowest SRCC, PLCC, and RMSE score, on the SIQA dataset. However, in the second attempt, with the use of the logistic optimization function, the proposed method outperformed the existing SIQA methods in terms of SRCC, PLCC, and RMSE.

### 3.6. Significance of Patch Size

During experimental evaluation, we perceived that parallax, blending, and blur error are difficult to capture at the patch level near smooth boundary regions such as white background, and are easily catchable in highly textured regions. In order to obtain optimal patch size, we conducted a series of experiments and evaluated the performance of our method across different patch sizes. The obtained results using different patch sizes are listed in Table 4. From the statistics presented in Table 4, it can be observed that using small patch sizes reduces the later quality estimation performance due to inaccurate localization of low texture regions at patch boundaries. In contrast, a very large patch size also negatively affected the overall performance of our system due to insufficient localization of stitching errors. Thus, we achieved a better tradeoff by choosing a suitable patch size for stitching induced error segmentation and overall quality estimation of the panoramic image.

### 3.7. Dominancy Analysis of Stitching Errors

**Figure 10:**
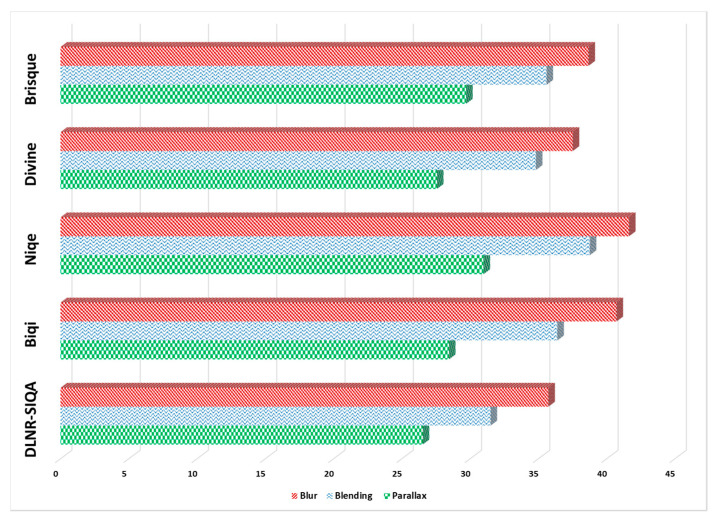
Dominancy analysis of three different stitching errors for panoramic image quality assessment.

To analyze the effect of specific stitching error in the quality reduction of panoramic images, we conducted an experimental study and investigated the dominancy of three different types of stitching errors, including parallax error, blending error, and blur error. For experimental purposes, we collected a total of 60 images (20 images per stitching error) and estimated the natural scene statistic of the selected test images using No-Reference IQA methods including BRISQUE (Blind/Reference Image Spatial Quality Evaluator) [53], DIIVINE (Distortion Identification-based Image Verity and Integrity Evaluation) [54], NIQE (Natural Image Quality Evaluator) [55], and BIQI (Blind Image Quality Indices) [56]. The main motivation behind the selection of these four methods was the fact that they do not compute the distortion specific features, i.e., blur distortion or blocking artifacts, but use scene statistics of locally normalized luminance coefficient of the image. The quality of the selected set of images was estimated using these four No-Reference IQA methods and computation of the average quality score of each method per stitching error. Besides, we also estimated the average quality score per stitching error using our proposed DLNR-SIQA method and compared the obtained score with other No-Reference IQA methods. The dominancy analysis of three different type of stitching error is depicted in Figure 10 where it can be observed that the quality score for each type of error ranges from 0 to 100. It is worth noticing that blur distorted images have the highest average quality score across all method, which shows the lowest dominancy of blur error/distortion on the quality of panoramic images. On the other hand, parallax images have the lowest average quality score through each method, showing the highest dominancy of parallax error/distortion on the perceptual quality of panoramic images. The blended distorted images have an average quality score between parallax and blur distortion, reflecting the average dominancy of the blended distortion on the quality of distorted images. Thus, the experimental study verified that parallax distortion has the highest dominancy, while blur distortion has the lowest dominancy on the quality of panoramic contents.

### 3.8. Limitations of Our Proposed System

Besides the effective performance for various types of stitching errors, there are certain limitations of the proposed method. Foremost, the proposed method is based on the Mask R-CNN network for error segmentation present in the panoramic images, where the size of these immersive contents ranges from 2k to 16k. As a result, the time complexity of the system is very high, thus limiting the performance of the proposed method in real-time. Further, in addition to the common stitching errors i.e., blur, parallax, and blending, there are other types of panoramic errors including ghosting effect, contrast variance, vignetting, and curved horizon, where the proposed method has limitations while dealing such errors in the panoramic images.

## 4. Conclusions and Future Work

Deep learning gained record-breaking performance recently in various fields of computer vision including object detection and segmentation, abnormal events detection, and activity recognition. Besides the trending fields of computer vision, errors analysis in images have recently been studied by researchers and enormous deep learning techniques have been proposed to automatically validate the quality of various traditional images. However, these methods are limited to evaluating the quality of traditional images and cannot be applied to panoramic images to evaluate the quality of their immersive contents. With these motivations, in this paper, we proposed a novel DLNR-SIQA framework to segment and extract three common types of stitching distorted regions (blend, parallax, and blur) present in panoramic images. In addition, we manually annotated three types of error using the Google Street View dataset and fine-tuned the Mask R-CNN for the segmentation and localization of the distorted regions. Finally, the areas of the distorted regions per pixel were measured to estimate the overall final quality of the panoramic image. To validate the performance of the proposed method, we used a set of well-known image segmentation performance evaluation metrics, including P, R, DSC, JI, mPA, mAP, and mAE, where our proposed method has dominance over the state-of-the-art methods. To further verify the generalization of our method, we also compared our method with existing SIQA methods using SRCC, PLCC, and RMSE measures. The obtained results revealed the effectiveness of our DLNR-SIQA framework, indicating that it is the most suitable aspirant for both visual inspection and quality assessment of panoramic images. Further, the proposed system can be used as part of the VR systems to segment and extract stitching distorted regions and measure the quality score of the immersive contents.

Currently, our proposed framework is only focused on a certain type of stitching distortion in panoramic images. In the future, we will extend this work to investigate the stitching induced distortions in 360° and VR videos. Further, this work can be intelligently merged with real-time stitching error detection and tracking.

## Figures and Tables

**Table 1:** Description of test sets used in experimental evaluation of our proposed framework.

Dataset	Number of Images	Image Resolution	Number of Patches	Image Compression Mode	Image Encoding Format
Test set A	300	256 × 256	1	Uncompressed	JPG
Test set B	160	4096 × 2048	128	Uncompressed	JPG

**Table 2:** The quantitative results obtained for distorted stitched patches and distorted panoramic images.

	Total Pixels	Distorted Pixels	Error-Free Pixels	Ratio of Error	Quality Score
**Quality Score for stitched patch images**					
Image 1	50,176	26,723	23,453	53.2585	46.7414
Image 2	301,015	2994	298,021	0.9946	99.0053
Image 3	50,176	1950	48,226	3.8863	96.1136
Image 4	274,924	4259	270,665	1.5491	98.4508
Image 5	50,176	1478	48,698	2.9456	97.0543
**Quality Score for 360° images**					
Image 1	8,388,608	44,795	8,343,813	0.5339	99.4661
Image 2	7,372,800	131,197	7,319,603	1.7794	98.2206
Image 3	7,786,489	61,054	7,725,435	0.7841	99.2159
Image 4	8,138,542	82,036	8,056,506	1.0079	98.9921
Image 5	7,865,136	83,198	7,781,938	1.0578	98.9422

**Table 3:** Comparison of our proposed DLNR-SIQA with different SIQA methods.

Method	SIQA Dataset			ISIQA Dataset		
	SRCC	PLCC	RMSE	SRCC	PLCC	RMSE
[34]	-	-	-	0.8724	0.8031	0.4417
[33]	0.7296	0.8572	0.3167	-	-	-
[31]	0.8431	0.9104	0.2378	-	-	-
DLNR-SIQA	0.8368	0.9056	0.2415	0.8193	0.8547	0.4181
DLNR-SIQA (Logistic function)	**0.8591**	**0.9367**	**0.2194**	**0.8463**	**0.8831**	**0.3952**

**Table 4:** Performance comparison of our proposed method across different patch sizes.

Experiments	Patch Resolution	Number of Patches	SRCC	PLCC	RMSE
1	64 × 64	2048	0.8753	0.8167	0.3342
2	128 × 128	512	0.8326	0.8629	0.2835
3	256 × 256	128	**0.8748**	**0.9421**	**0.2157**
4	512 × 512	32	0.8514	0.9176	0.2491
